# Evaluation of Antioxidant Activity and Treatment of Eczema by Berberine Hydrochloride-Loaded Liposomes-in-Gel

**DOI:** 10.3390/molecules29071566

**Published:** 2024-03-31

**Authors:** Si Shen, Xiaobo Qu, Yinyin Liu, Mengmeng Wang, Haifeng Zhou, Hongmei Xia

**Affiliations:** 1 College of Pharmacy, Anhui University of Chinese Medicine, No. 350, Long Zi Hu Road, Hefei 230012, China; shensi@stu.ahtcm.edu.cn (S.S.); xiaoboqu2022@163.com (X.Q.); lyy123@stu.ahtcm.edu.cn (Y.L.);; 2 Drug Advanced Research Institute of Yangtze Delta, Nantong 226100, China

**Keywords:** berberine hydrochloride, liposomes, liposomes-in-gel, eczema, antioxidant capacity

## Abstract

In this paper, berberine hydrochloride-loaded liposomes-in-gel were designed and developed to investigate their antioxidant properties and therapeutic effects on the eczema model of the mouse. Berberine hydrochloride-liposomes (BBH-L) as the nanoparticles were prepared by the thin-film hydration method and then dispersed BBH-L evenly in the gel matrix to prepare the berberine hydrochloride liposomes-gel (BBH-L-Gel) by the natural swelling method. Their antioxidant capacity was investigated by the free radical scavenging ability on 2,2-diphenyl-1-picrylhydrazyl (DPPH) and H_2_O_2_ and the inhibition of lipid peroxides malondialdehyde (MDA). An eczema model was established, and the efficacy of the eczema treatment was preliminarily evaluated using ear swelling, the spleen index, and pathological sections as indicators. The results indicate that the entrapment efficiency of BBH-L prepared by the thin-film hydration method was 78.56% ± 0.7%, with a particle size of 155.4 ± 9.3 nm. For BBH-L-Gel, the viscosity and pH were 18.16 ± 6.34 m Pas and 7.32 ± 0.08, respectively. The cumulative release in the unit area of the in vitro transdermal study was 85.01 ± 4.53 μg/cm^2^. BBH-L-Gel had a good scavenging capacity on DPPH and H_2_O_2_, and it could effectively inhibit the production of hepatic lipid peroxides MDA in the concentration range of 0.4–2.0 mg/mL. The topical application of BBH-L-Gel could effectively alleviate eczema symptoms and reduce oxidative stress injury in mice. This study demonstrates that BBH-L-Gel has good skin permeability, excellent sustained release, and antioxidant capabilities. They can effectively alleviate the itching, inflammation, and allergic symptoms caused by eczema, providing a new strategy for clinical applications in eczema treatment.

## 1. Introduction

Berberine hydrochloride (BBH), characterized by yellow needle-like crystals, is a quaternary isoquinoline alkaloid primarily derived from Huanglian (Coptidis Rhizoma) [1]. Huanglian (Coptidis Rhizoma) contains a variety of chemical components, including alkaloids, lignans, flavonoids, acidic compounds, and essential oils [2]. Berberine is classified as a flavonoid, a group of compounds that has garnered significant attention in the pharmaceutical and healthcare sectors in recent years [3]. Flavonoids, sourced mainly from fruits, vegetables, and cereals, have demonstrated various pharmacological properties in studies, such as antibacterial, anti-inflammatory, antioxidant, anticancer, and antiarrhythmic effects [4].

Recent domestic and international research has highlighted the potential of BBH as a valuable active ingredient in medicinal plants. BBH has demonstrated the ability to expedite wound healing, alleviate inflammation and swelling, and facilitate the growth of granulation tissue for wound repair [5,6]. The linperlisib studied by our research team is also a good anti-inflammatory, antioxidant, and anti-tumor drug. Moreover, BBH exhibits anti-inflammatory properties by inhibiting inflammatory cell infiltration, modulating signaling pathways such as MAPK and NF-κB, and suppressing the secretion and expression of key factors like IL-1β, IL-6, IL-17, TNF-α, and INF-γ, offering therapeutic benefits across a spectrum of diseases [7,8]. BBH is also effective in scavenging reactive oxygen species, deactivating electron reduction products such as O^2−^, OH^−^, H_2_O_2_, and NO, enhancing antioxidant enzyme activity, and minimizing lipid peroxidation [9]. Notably, BBH has shown efficacy in treating various dermatological conditions, with remarkable results observed when the BBH aqueous solution is applied to eczema. Research findings have shown that BBH significantly improved DNFB-induced contact dermatitis in rat models [10].

Eczema is an inflammatory skin disease influenced by various internal and external factors [11]. The condition is characterized by erythema, papules, blisters, and desquamation, often accompanied by oozing [12]. BBH exhibits the potential to suppress oxidative stress production and possesses substantial anti-inflammatory properties for treating eczema. The incidence of eczema is progressively increasing annually, attributed to factors like environmental changes, heightened life pressures, and alterations in dietary habits. In the clinical management of eczema, oral antihistamines, antibiotics, and glucocorticoid ointments are commonly prescribed [13]. Usually, oral drug delivery is hindered by gastrointestinal absorption stimulation, while conventional topical skin dosage forms are impeded by the stratum corneum barrier, limiting their therapeutic efficacy. Nanocarriers present a promising avenue for enhancing skin drug delivery by effectively increasing drug permeability and improving dermal retention properties [14]. Liposomes, typically composed of phospholipids and cholesterol, serve as valuable carriers. Phospholipids act as penetration enhancers, facilitating drug penetration into the stratum corneum and epidermal lipids [15,16,17]. Furthermore, liposomes aid in enhancing local drug accumulation in the skin by minimizing systemic absorption. Liposomes, being amphiphilic, serve as versatile carriers capable of encapsulating various drug molecules for targeted delivery to skin tissue [18]. Hydrophilic drugs like BBH are typically housed within the internal aqueous phase of liposomes. Despite their efficacy, liposomes in suspension form exhibit a limited residence time on the skin. Gels, characterized by a hydrophilic nature and a three-dimensional network structure comprising natural or synthetic macromolecular polymers, possess excellent adhesive properties. The high water content in gels enhances cuticle hydration, thereby altering barrier permeability [19,20]. Liposome gel amalgamates the benefits of liposomes and hydrogels, emerging as a versatile vehicle for skin therapy [21]. This formulation addresses the stability issues concerning traditional liposomes related to pH and ionic strength, showcasing superior efficiency in delivering hydrophilic drugs compared to conventional gels. The liposomes-in-gel formulation can prolong the contact time between the drug and the skin, increase the concentration of the drug at the site of administration, change the permeability of tissues to promote drug absorption, and significantly improve the therapeutic index of the drug [22].

**Figure 1:**
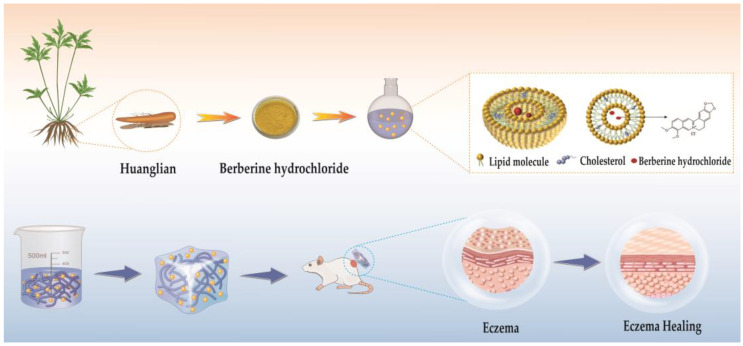
The preparation process of berberine hydrochloride liposomes-gel (BBH-L-Gel) and its role in the treatment of eczema.

Therefore, the aim of this paper was to prepare BBH-L-Gel and to investigate its antioxidant properties and therapeutic effects on eczema (Figure 1). Liposomes-in-gel represent an innovative dermal dosage form designed to facilitate a sustained drug release for prolonged efficacy, offering a fresh approach for applying BBH in eczema treatment.

## 2. Results and Discussion

### 2.1. Encapsulation Rate, Particle Size, and Potential of Liposomes

The encapsulation efficiency of BBH-L, as measured post-centrifugation, was 78.56% ± 5.70. The liposomes formulated using the thin-film hydration technique at a soya-bean-lecithin-to-cholesterol ratio of 3:1 exhibited a notable encapsulation efficacy for berberine hydrochloride, effectively enhancing its solubility.

The average particle size of blank liposomes (B-L), as measured by a particle size analyzer, was 130.6 ± 10.8 nm, with a zeta potential of −31.2 ± 1.6 mV. In contrast, the average particle size of the BBH-L was 155.4 ± 9.3 nm, with a zeta potential of −33.6 ± 2.7 mV. These findings suggest that liposomes prepared using the thin-film hydration method exhibit a small particle size, potentially enhancing their ability to penetrate the skin. The negative zeta potential of the liposomes indicates their stability, while the zeta potential of drug-containing liposomes showed little variation compared to blank liposomes, suggesting that berberine hydrochloride does not significantly alter the potential.

### 2.2. Viscosity and pH of Liposomes-in-Gel

The viscosity of the B-Gel, B-L-Gel, BBH-Gel, and BBH-L-Gel measured in the experiment was 8.25 ± 0.76, 15.39 ± 0.18, 9.53 ± 0.88, and 18.16 ± 6.34 m Pas, respectively. The pH values of B-Gel, B-L-Gel, BBH-Gel, and BBH-L-Gel were 7.11 ± 0.14, 7.28 ± 0.12, 7.27 ± 0.06, and 7.32 ± 0.08. The results indicated that the viscosity of the blank liposome gel (B-L-Gel) was higher than that of the blank gel (B-Gel), while the drug-loaded gel exhibited a viscosity similar to that of B-Gel. The addition of 0.5 mg/mL BBH did not alter the viscosity of the sodium alginate gel. The pH levels of the gel and liposomes-in-gel closely matched that of the PBS solution (pH = 7.4), suggesting that berberine hydrochloride and liposomes had a minimal impact on the gel’s pH. The pH value of the gel formulations commonly used for skin drug delivery is around 7.0, and BBH-L-Gel meets the requirement. For transdermal drug delivery, liposomes have high fluidity, which is not conducive to prolonged retention on the local skin. On the other hand, gels have good adhesiveness, can closely adhere to the lesion site, and are easily absorbed by the affected area. BBH-L-Gel has a moderately suitable viscosity, good spreadability, and easy application to the skin and is easy to wash off. Sodium alginate was added to the liposome suspension, which was naturally dissolved to obtain a fine-textured liposome gel, in which the liposomes could be uniformly dispersed, the pH showed weak alkaline, and the viscosity was moderate, with a certain degree of adhesion to the skin, which was suitable for dermal administration. For skin administration, formulations with some viscosity and a pH close to 7.4 are preferred, making BBH-L-Gel the most suitable choice. Sodium alginate is known for being non-irritating, biocompatible, and capable of forming three-dimensional cross-linked gels. It directly targets macrophages to facilitate wound healing, making it versatile for various biomedical applications and serving as a platform for diverse forms of drug delivery [23].

### 2.3. In Vitro Release Study

**Figure 2:**
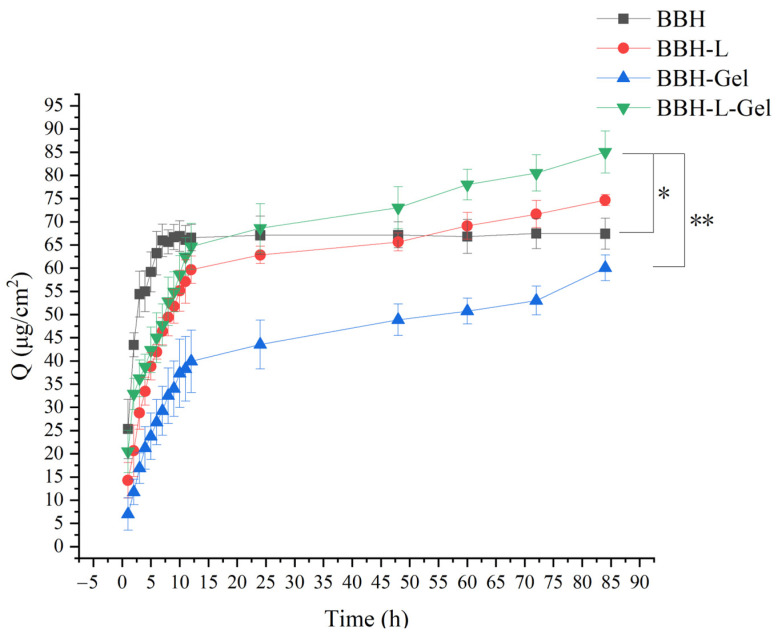
Cumulative release per unit area of berberine hydrochloride across skin in vitro. Each value represents the mean ± standard deviation, *n* = 3. * *p* < 0.05, ** *p* < 0.01. In the figure, BBH represents berberine hydrochloride; BBH-L represents berberine hydrochloride-liposomes; BBH-Gel represents berberine hydrochloride-gel; BBH-L-Gel represents berberine hydrochloride liposomes-gel.

The in vitro cumulative release of BBH per unit area through the skin is illustrated in Figure 2. At 84 h, the cumulative release of BBH-L-Gel per unit area reached 85.01 ± 4.53 μg/cm^2^, surpassing that of the BBH, BBH-L, and BBH-Gel groups. To traverse the skin, drugs must permeate the epidermis and dermis. The epidermis is divided into the stratum corneum, transparent layer, granular layer, spinous layer, and basal layer from the outside to the inside. The dermis is a connective tissue located under the epidermis and consists of fibers, matrix, cells, blood vessels, and nerves [24]. Thus, the drugs exist in large quantities in the skin and act through blood vessels [25]. The water solubility and skin permeability of berberine hydrochloride restricted its skin usability, and combining it with a fat-soluble complex to change its solubility will increase skin permeability [26]. Because liposomes have a similar lipid structure as skin, which is beneficial for drugs penetrating through skin, the cumulative release amount of BBH-L and BBH-L-Gel per unit area was high, and the adhesion of the gel to the skin can enhance the release of drugs. The release rate of BBH was faster, which showed that liposomes and liposomes-in-gel had a good sustained and controlled release effect on berberine hydrochloride and could prolong the action time of the drug. Studies have shown that proniosome-gel can enhance the penetration of berberine into the skin and reduce inflammation and pain in arthritis rats [26].

### 2.4. Results of Antioxidant Activity of BBH-L-Gel

**Figure 3:**
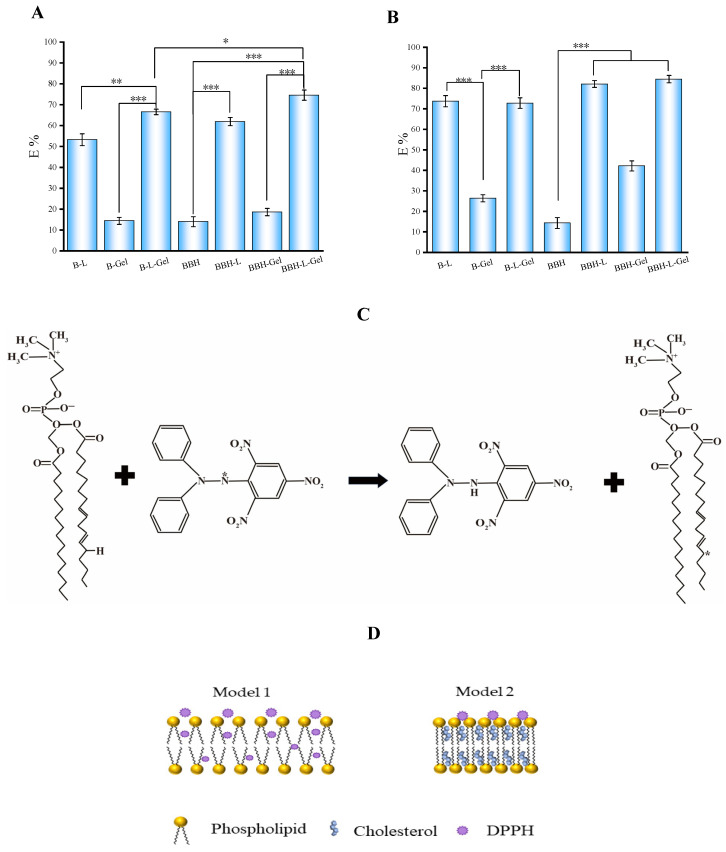
Free radical scavenging rate of berberine hydrochloride-loaded preparations: (**A**) DPPH free radical; (**B**) H_2_O_2_. In the figure, B-L represents blank liposomes; B-Gel represents blank gel; B-L-Gel represents blank liposomes-gel; BBH represents berberine hydrochloride; BBH-L represents berberine hydrochloride-liposomes; BBH-Gel represents berberine hydrochloride-gel; BBH-L-Gel represents berberine hydrochloride liposomes-gel. The results are all expressed as the mean ± standard deviation, *n* = 3. * *p* < 0.05, ** *p* < 0.01, *** *p* < 0.001. Interaction between phospholipid molecules and DPPH free radicals: (**C**) reaction of the DPPH free radical and phospholipid; (**D**) the schematic diagram of cholesterol-free (model 1) and cholesterol-containing (model 2) liposomes under the oxidation of DPPH free radicals.

The results of the scavenging rate on DPPH radicals and H_2_O_2_ by each group of preparations are shown in Figure 3A,B.The scavenging capacity of BBH on DPPH radicals and H_2_O_2_ was low, while the scavenging rate on DPPH radicals and H_2_O_2_ by preparations loaded with BBH was higher than that of the BBH group, suggesting a synergistic antioxidant effect of the drug with the preparations [27]. The study examined the scavenging capacity of linperlisib and its tablets on DPPH free radicals. The findings demonstrated that a concentration of 20 mg/mL of linperlisib effectively scavenged 74.6 ± 1.26% of DPPH free radicals, highlighting its significant antioxidant potential. Antioxidant defense mechanisms primarily aim to counteract the accumulation of excess reactive oxygen species (ROS) and uphold redox homeostasis. The groups containing liposomes showed good clearance, indicating that liposomes have a significant antioxidant effect [28,29]. Liposomes consist of a certain amount of cholesterol filled in a phospholipid bilayer, and hydrogen bonds are formed between the hydroxyl groups of cholesterol and the hydrophilic ends of phospholipids to form a stable system; when the concentration of phospholipids increases, the phospholipid bilayer is unstable, and its unsaturated fatty acid chains, as well as phosphoric acid groups, may share electrons to react with DPPH radicals [30]. The degree of unsaturation of fatty acids on the phospholipid structure is positively correlated with its antioxidant activity. The reaction equation of soybean phospholipids with DPPH is shown in Figure 3C. The effect of the cholesterol concentration on phospholipids varies in effect, with lower concentrations interfering more strongly between the polar head groups of phospholipids, dispersing the phospholipid bilayer and making it easier for the DPPH radical to enter and react within the phospholipid molecule, with high concentrations of cholesterol tending to act on the hydrocarbon chains in the tails of the phospholipids to make the overall binding more tightly, and with the hardening of the cholesterol between the bilayers [31] decreasing the rate of reaction of the DPPH radicals. The action of DPPH on liposomes is shown schematically in Figure 3D. The strong oxidative action of H_2_O_2_ can cause irreversible damage to membranes, and cholesterol also undergoes redox reactions with it. Consequently, increased cholesterol concentrations also increased the clearance of H_2_O_2_ from liposome membranes.

The clearance rate of the liposomes-in-gel group was higher than that of the liposome group, probably due to the encapsulation of liposomes by the sodium alginate gel network to enhance their stability and reduce the autoxidation of phospholipids. Sodium alginate itself also has a certain antioxidant effect; research showed that in a certain mass concentration range, sodium alginate solution could show good antioxidant effects [32]. The scavenging rate on H_2_O_2_ by sodium alginate was higher than the scavenging on DPPH radicals, and the strong oxidizing properties of H_2_O_2_ may have damaged its molecular structure [33]. Overall, both BBH and BBH-L-Gel have good antioxidant properties, and they complement each other and are suitable for the treatment of skin diseases in a state of inflammation such as eczema.

### 2.5. Therapeutic Effect of BBH-L-Gel on Eczema

#### 2.5.1. Weight Changes of Mice

**Figure 4:**
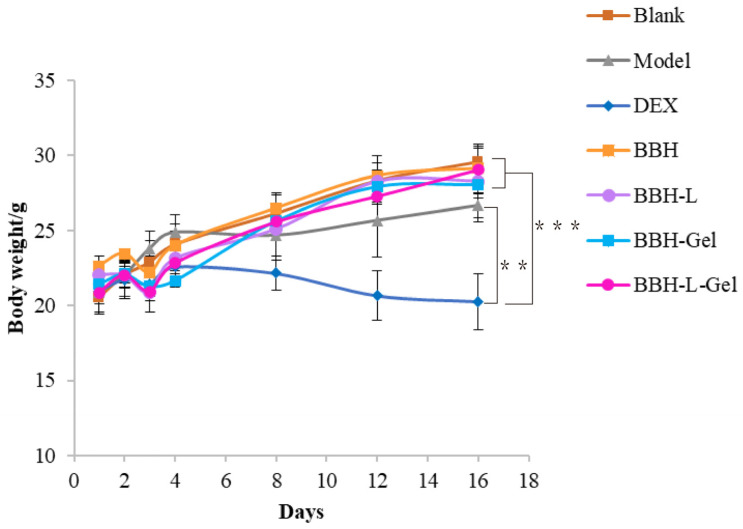
The weight changes of mice. Each value represents the mean ± standard deviation, *n* = 6. ** *p* < 0.01, *** *p* < 0.001. In the figure, BBH represents berberine hydrochloride; BBH-L represents berberine hydrochloride-liposomes; BBH-Gel represents berberine hydrochloride-gel; BBH-L-Gel represents berberine hydrochloride liposomes-gel.

The weight of mice is closely associated with their health status. As depicted in Figure 4, the mouse weight rises with prolonged drug exposure. Interestingly, the DEX group exhibits weight loss, potentially attributed to glucocorticoid side effects. Consequently, this reversal serves as evidence that our drug treatment not only demonstrates efficacy but also mitigates the occurrence of adverse reactions [34].

#### 2.5.2. Thickness Changes in Skin in Mice

**Figure 5:**
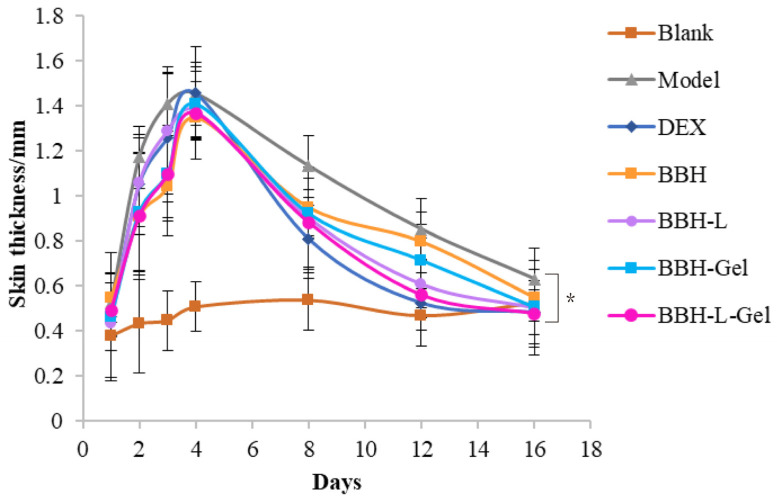
Skin thickness on the back of mice. Each value represents the mean ± standard deviation, *n* = 6. * *p* < 0.05. In the figure, BBH represents berberine hydrochloride; BBH-L represents berberine hydrochloride-liposomes; BBH-Gel represents berberine hydrochloride-gel; BBH-L-Gel represents berberine hydrochloride liposomes-gel.

The skin undergoes repeated inflammation when the symptoms of eczema appear in the skin. The epidermis layer will thicken, and the stratum corneum, which should be metabolized normally, is accelerated because of the inflammation, resulting in a lot of keratin that is too late to fall off, half of which sticks to the skin and half lifts up, which is the manifestation of peeling or scaling that we see clinically [35,36]. The change in skin thickness on the back of mice is shown in Figure 5. Except for the blank group, the skin thickness of each group showed a trend of increasing at first and then decreasing. In general, the skin damage caused by DNCB became thicker. With the self-healing of mice and after medication, the skin damage gradually improved, scabs fell off, and the skin thickness decreased.

#### 2.5.3. EASI Score

**Figure 6:**
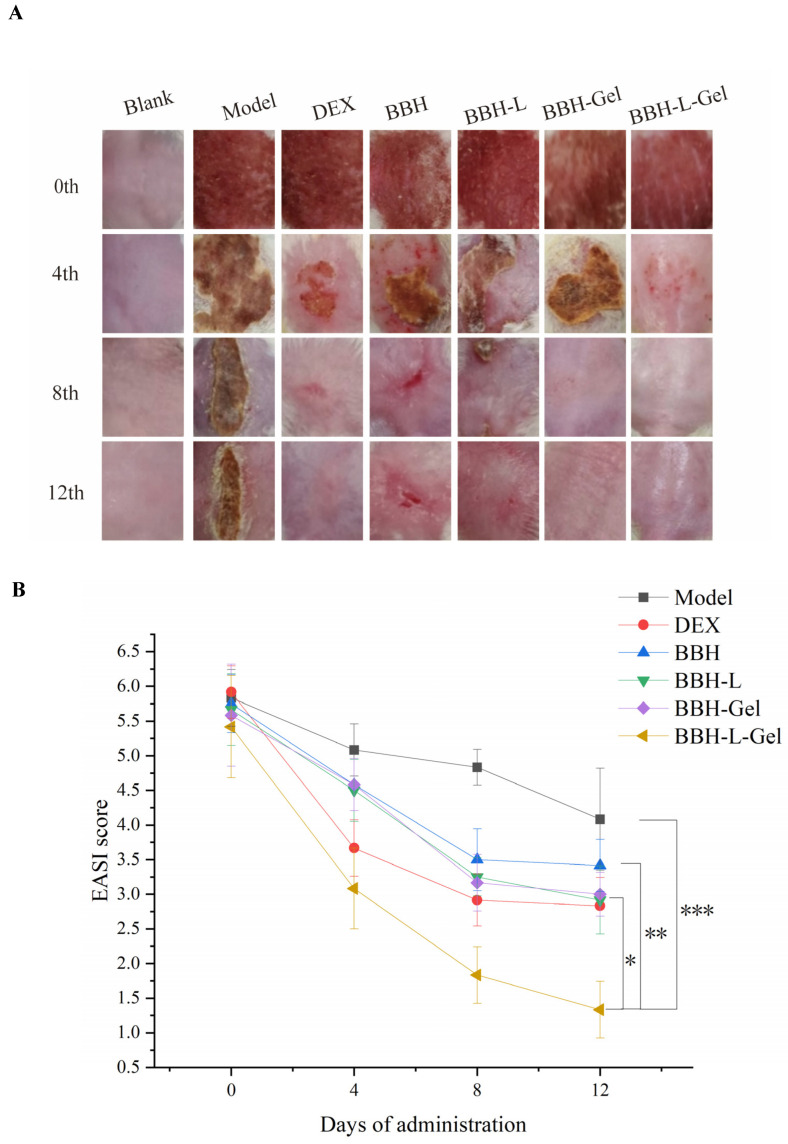
(**A**) Photographs of skin lesions on the back of mice after continuous administration for 12 days. (**B**) Scoring results of skin lesions in mice. Each value represents the mean ± standard deviation, *n* = 6. * *p* < 0.05, ** *p* < 0.01, *** *p* < 0.001. In the figure, BBH represents berberine hydrochloride; BBH-L represents berberine hydrochloride-liposomes; BBH-Gel represents berberine hydrochloride-gel; BBH-L-Gel represents berberine hydrochloride liposomes-gel.

In the blank group, the skin on the back of mice was reddish, delicate, tender, and soft, and the skin texture is clearly visible (Figure 6A). Two days after sensitization with DNCB, the skin on the back of mice was obviously damaged, and symptoms such as redness, scabbing, scaling, and thickening appeared, which conformed to the diagnosis symptoms of eczema, indicating that the eczema model was successfully reproduced. Figure 6B shows the skin lesion score of mice. Before taking the medicine, except for the blank group, the degree of the back skin lesions of mice in each group was the same, and there was no significant difference in the skin lesions score (*p* > 0.05). With the treatment of DEX and berberine hydrochloride preparation, the skin lesions on the back of mice improved obviously, and the skin lesion score began to decline after 4 days of administration. After 8 days, the skin lesion score of mice decreased significantly (*p* < 0.05). After 12 days of medication, the back skin of the BBH group was still red, swollen, and damaged, BBH-L and BBH-Gel had some papules, DEX and BBH-L-Gel had only a little erythema, and BBH-L-Gel had a lower skin lesion score than DEX, which indicated that BBH-L-Gel had a better effect on reducing skin lesions than DEX. In the model group, the wounds of mice had a self-healing process without medication, but compared with the medication group, the recovery process was slower.

#### 2.5.4. Scratching Times of Mice

**Figure 7:**
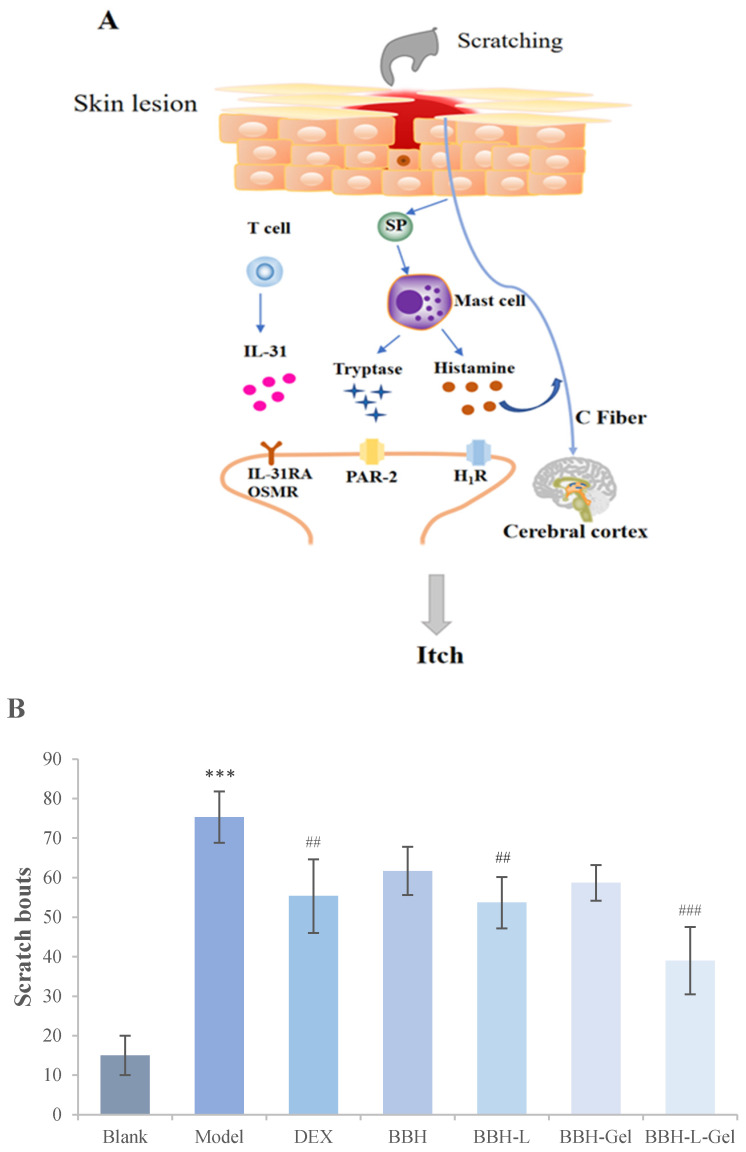
(**A**) Signal pathway of itching in eczema; (**B**) Scratching bouts of mice after drug treatment. The results are all expressed as the mean ± standard deviation, *n* = 6. *** *p* < 0.001 versus blank; ^##^ *p* < 0.01, ^###^ *p* < 0.001 versus model. In the figure, BBH represents berberine hydrochloride; BBH-L represents berberine hydrochloride-liposomes; BBH-Gel represents berberine hydrochloride-gel; BBH-L-Gel represents berberine hydrochloride liposomes-gel.

Itching is one of the main clinical symptoms of eczema [37]. Severe itching will lead to scratching, which will lead to the release of neuropeptides such as SP and a large number of inflammatory factors such as IL-31, which will stimulate mast cells to release histamine or directly stimulate C-Fiber endings to induce skin itching [38,39]. The signal chart of eczema itching is shown in Figure 7A, which shows the results of the scratching times of mice after drug treatment. Compared with the blank group, the repeated application of DNCB in the model group prominently increased the scratching times of mice, indicating that DNCB sensitization caused back itching in mice. After 12 days of the administration, the scratching times of mice in the administration group decreased, and the scratching times of the BBH-L-Gel group were the lowest, which indicated that the cumulative effect of BBH-L-Gel was better than that of the other four groups.

#### 2.5.5. Ear Swelling

**Figure 8:**
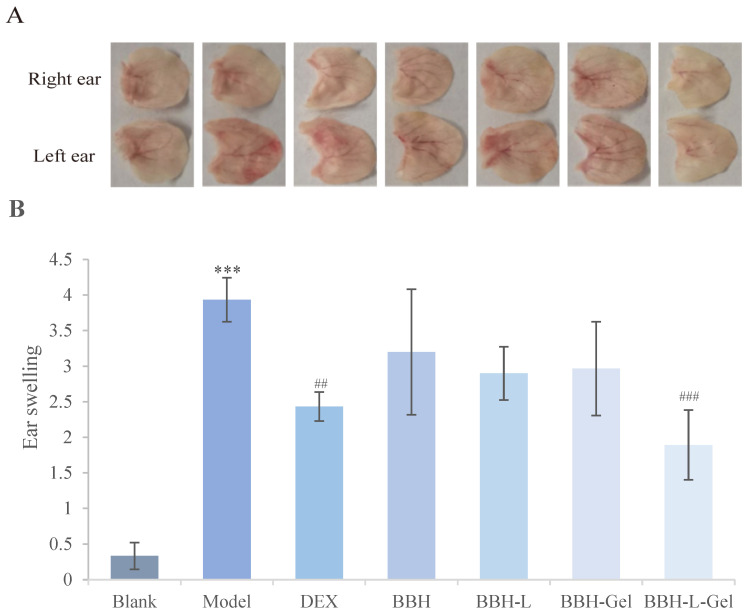
(**A**) Photographs of mice’s ears after DNCB sensitization. (**B**) Ear swelling of mice. The results are all expressed as the mean ± standard deviation, *n* = 3. *** *p* < 0.001 versus blank; ^##^ *p* < 0.01, ^###^ *p* < 0.001 versus model. In the figure, BBH represents berberine hydrochloride; BBH-L represents berberine hydrochloride-liposomes; BBH-Gel represents berberine hydrochloride-gel; BBH-L-Gel represents berberine hydrochloride liposomes-gel.

The left ear sensitized by DNCB will show obvious redness and swelling (Figure 8A), which will recover after drug treatment. Figure 8B shows the results of ear swelling in mice after drug treatment. Compared with the blank group, the ear swelling in the model group was significantly increased (*p* < 0.05). In addition, the degree of ear swelling can be representative of inflammation in mice. The degree of ear swelling in the model group was larger, indicating that the mice had obvious inflammation after DNCB induction [40,41]. Compared with the model group, BBH, BBH-L, and BBH-Gel had certain inhibitory effects on the ear swelling of mice, and the ear swelling of the positive control group and BBH-L-Gel group was obviously reduced (*p* < 0.05). The results indicate that inflammation and swelling in the ears of mice in the BBH-L-Gel treatment group were significantly inhibited, showing a good therapeutic effect. Therefore, the improvement of the berberine hydrochloride formulation is of great significance.

#### 2.5.6. Weight Ratio of the Spleen to the Body

**Figure 9:**
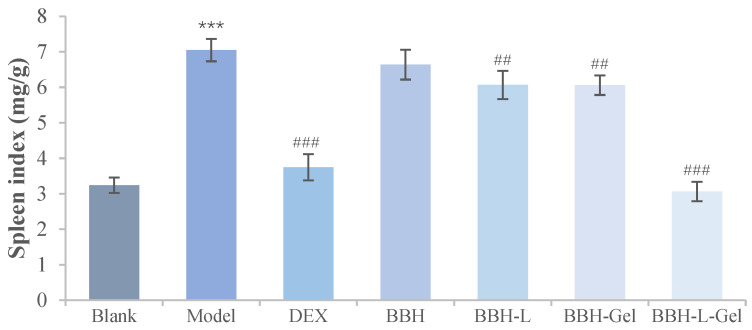
Spleen index result of mice. The results are all expressed as the mean ± standard deviation, *n* = 6. *** *p* < 0.001 versus blank; ^##^ *p* < 0.01, ^###^ *p* < 0.001 versus model. In the figure, BBH represents berberine hydrochloride; BBH-L represents berberine hydrochloride-liposomes; BBH-Gel represents berberine hydrochloride-gel; BBH-L-Gel represents berberine hydrochloride liposomes-gel.

Figure 9 showed that the mice induced by DNCB had obvious splenomegaly, and the spleen index was significantly increased compared with the blank group (*p* < 0.05). After drug treatment, the spleen weight of mice in the DEX, BBH, BBH-L, BBH-Gel, and BBH-L-Gel groups decreased. Among them, DEX and BBH-L-Gel had the best effect on reducing the spleen index. The spleen contains a large number of immune cells, and it is the largest immune organ in the body. The spleen index is an important index for reflecting the immune function of the body [42]. Eczema will lead to abnormal immune function in mice, which will lead to an increase in the spleen index [43]. Berberine hydrochloride could reduce the spleen index increased by eczema, and it had the same curative effect as DEX. The results showed that its mechanism might be related to immune regulation [44].

#### 2.5.7. Determination of Oxidative Stress Caused by Eczema

**Figure 10:**
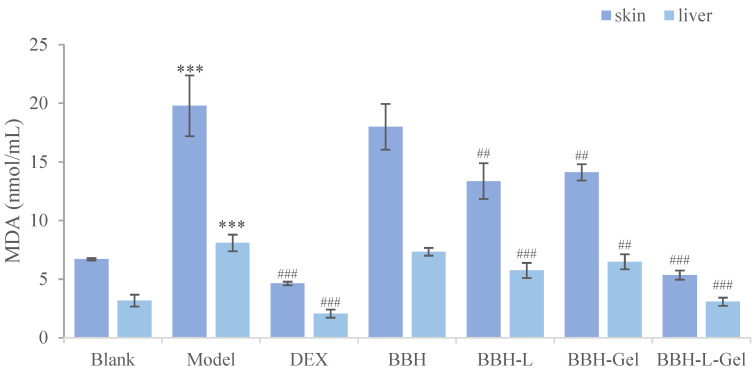
Effect of BBH on MDA in the skin and liver of mice. The results are all expressed as the mean ± standard deviation, *n* = 6. *** *p* < 0.001 versus blank; ^##^ *p* < 0.01, ^###^ *p* < 0.001 versus model. In the figure, BBH represents berberine hydrochloride; BBH-L represents berberine hydrochloride-liposomes; BBH-Gel represents berberine hydrochloride-gel; BBH-L-Gel represents berberine hydrochloride liposomes-gel.

MDA is the product of oxidative stress, which can reflect the degree of injury to the body [45,46]. The results of the MDA determination of mice in each group were shown in Figure 10. According to the absorbance results of MDA, the oxidation degrees of the skin and liver of mice were evaluated, respectively [47]. Compared with the blank group, the MDA values of the skin and liver were significantly increased, indicating that eczema caused oxidative damage in mice. Furthermore, the MDA value in the positive control group and BBH-L-Gel group decreased significantly, and DEX and BBH-L-Gel could effectively reduce the oxidative stress caused by eczema. The effect of reducing MDA was not significant in the BBH, BBH-L, and BBH-Gel groups, and it is speculated that the main reason is the poor adhesion of berberine hydrochloride and liposomes to the skin and the low transdermal efficiency of the drug. Liposomes-in-gel can increase skin adhesion, and liposomes have good permeability and biocompatibility [48,49], thereby enhancing the therapeutic effect of berberine hydrochloride. A lot of studies have shown that berberine hydrochloride has a certain antioxidant effect [50,51], which can effectively reduce the damage of oxidative stress in the body and then play a role in the treatment of eczema and anti-inflammatory factors via the liposomes-in-gel as its delivery.

## 3. Materials and Methods

### 3.1. Materials

Berberine hydrochloride (purity > 98%) was obtained from Xi’an Ryan Biotechnology Co., Ltd. (Xi’an, China). Soy lecithin, cholesterol, and sodium alginate were obtained from Tianjin Guangfu Fine Chemical Research Institut (Tianjin, China). Ethanol absolute (AR), acetone, and olive oil were purchased from Shanghai RichJoint Chemical Reagents Co., Ltd. (Shanghai, China). 2,4-dinitrochlorobenzene was purchased from the Xiya reagent company (Shandong, China). Thiobarbituric acid (TBA) was purchased from Shanghai Yuanye Biology Science and Technology Co., Ltd. (Shanghai, China). Trichloroacetic acid (TCA) (AR) was purchased from Sinopharm Chemical Reagent Co., Ltd. (Shanghai, China).

### 3.2. Preparation of BBH-L-Gel

BBH-L were prepared using the thin-film dispersion method. First, 0.3 g of soy lecithin and 0.1 g of cholesterol were weighed and 5 mL of anhydrous ethanol was added, and the flask was rotated after the complete dissolution by ultrasound so that a thin film was formed within the walls as the ethanol evaporated. The BBH-L suspension was prepared by adding 0.5 mg/mL BBH solution and hydrating it for 1 h at 40 °C. BBH-L-Gel can be obtained by taking 10 mL of the prepared BBH-L suspension, adding 0.1 g of sodium alginate powder, placing it on a magnetic stirrer and mixing it well, and leaving it overnight to make the gel swell sufficiently, and it can be obtained. Berberine hydrochloride-gel (BBH-Gel) was prepared with BBH solution in the same way.

### 3.3. Characterization of Liposomes-in-Gel

#### 3.3.1. Encapsulation Efficacy and Particle Size of Liposomes

The encapsulation efficiency (EN) of liposomes was determined by centrifugation. A total of 2 mL of BBH-L was taken in a centrifuge tube and centrifuged at 12,000 r/min for 20 min, 0.5 mL of the supernatant was sucked into a 10 mL volumetric flask, and PBS solution was added to a constant volume. A proper amount of the solution was taken, the absorbance at 345 nm was measured with an ultra-violet spectrophotometer (UV-1000, AOE Instrument Co., Ltd., Shanghai, China), and the free drug concentration (C_free_) was calculated. In total, 1 mL BBH-L was taken in a 10 mL volumetric flask. Absolute ethanol was added to demulsify and fix the volume, and it was dissolved in a water bath ultrasound for 10 min. A proper amount of the solution was taken to measure the absorbance and calculate the total drug concentration (C_total_). The entrapment efficiency was calculated according to the following Formula (1):

(1) $$\mathrm{EN}\%=\frac{C_{\mathrm{total}}-\;C_{\mathrm{free}}}{C_{\mathrm{total}}}$$

In the formula, “C_total_” is the concentration of the drug successfully encapsulated in the liposome and “C_free_” is the concentration of a drug that has not yet been successfully encapsulated in liposomes.

After a proper amount of liposome solution was taken and diluted ten times, the particle size and zeta potential of the liposomes were determined using dynamic light scattering (DLS) (Malvern Instruments, Malvern, UK). Each subject was tested in triplicate.

#### 3.3.2. Measurement of the Viscosity and pH of BBH-L-Gel

The gel samples were diluted five times, respectively, and the temperature was set at 25 °C. The viscosity of each gel and liposome-in-gel was measured using an Austrian viscometer, and the time was recorded. The pH values of the gels and liposome gels were determined using a pH meter (Leici PHSJ-4A, Shanghai, China), and the average values were calculated by three parallel measurements.

### 3.4. In Vitro Study of Transdermal Release

The mice were anesthetized, and the hair on the back of the mice was removed with an electric shaver and cleaned with depilatory cream. The skin was taken off and laid flat on a glass plate with the stratum corneum facing down, subcutaneous adipose tissue and adhesions were removed with tweezers, and it was repeatedly rinsed with normal saline for later use.

In order to evaluate the transdermal ability of BBH-L-Gel, a Franz diffusion cell was used for the transdermal study in vitro. The skin was fixed on the diffusion pool, with the cuticle facing the drug supply and the dermis facing the receiving solution. In total, 1 mL of BBH or its preparations was put into the supply pool, the receiving pool was filled with PBS solution (pH = 7.4), and the experimental temperature was set at 37 ± 0.5 °C. Samples were taken at 1 h, 2 h, 3 h, 4 h, 5 h, 6 h, 7 h, 8 h, 9 h, 10 h, 11 h, 12 h, 24 h, 36 h, 48 h, 60 h, 72 h, and 84 h, respectively, and the same amount of PBS solution was added to the receiving pool after each sampling. The absorbance of the sample was measured by an ultraviolet spectrophotometer. The cumulative transdermal quantities (Q) per unit area of the drug could be calculated by the following Formula (2):

(2) $$Q=\frac{C_{n}\times V+\sum_{i=1}^{n-1}\;C_{i}\times 2}{S}$$

In the formula, “C_n_” is the drug concentration measured at the n-th sampling point, “C_i_” is the drug concentration measured at the i-th (i = n − 1) sampling point, “V” is the total volume of the released liquid, “2” is the sampling volume and “S” is the release area (cm^2^).

### 3.5. Determination of the Antioxidant Activity of the BBH-L-Gel

#### 3.5.1. Determination of Scavenging Ability on a DPPH Radical

The DPPH free radical is deemed a highly stable chemical substance with a structure incorporating a lone pair of electrons. When combined with antioxidant substances, electron transfer takes place, resulting in the reduction in the DPPH radical to DPPH-H and the alteration of the color from deep purple to light yellow. The DPPH assay is recognized as one of the most effective and widely utilized methods in antioxidant activity testing. The DPPH methodology has been slightly modified from previous reports [52]. In total, 0.2 mmol/L DPPH solution was prepared, and berberine hydrochloride formulation groups were diluted 10-fold as a sample solution. A total of 2 mL of the sample solution was mixed with 1 mL of the DPPH solution, reacted in the dark for 30 min, and adjusted to zero with anhydrous ethanol, and absorbance was measured at 517 nm. PBS solution was used in place of the sample solution in the blank control group, and anhydrous ethanol was used in place of the DPPH solution in the sample control group. The DPPH radical scavenging rate can be calculated by Formula (3), where A_s_ is the absorbance of the sample group, A_c_ is the absorbance of the sample control group, and A_0_ is the absorbance of the blank group.

(3) $$E\%=\left(1\;-\;\frac{A_{s}\;-A_{c}}{A_{0}}\right)\times 100\%$$

#### 3.5.2. Determination of H_2_O_2_ Scavenging Ability

The H_2_O_2_ methodology has been slightly modified from previous reports [53]. In total, 40 mmol/L H_2_O_2_ solution was prepared. Then, 0.6 mL of the sample solution and 1.8 mL of the H_2_O_2_ solution were taken in a centrifuge tube and mixed well, and after 10 min of reaction, the absorbance was measured at 230 nm after zeroing with distilled water. The blank group and sample control group were replaced with 0.6 mL of the PBS solution and distilled water, respectively, and the formula for calculating the H_2_O_2_ clearance was the same as that presented above (3).

### 3.6. Pharmacodynamic Study on Eczema

#### 3.6.1. Establishment of an Eczema Model in Mice

In total, 42 female mice (N = 6) were randomly divided into the blank group, model group, positive control group (DEX), BBH group, BBH-L group, BBH-Gel group, and BBH-L-Gel group. On the day before the experiment, depilatory cream was used to remove the back hair of mice, and the exposed area was 2 cm × 2 cm. Except for the blank group, all the other groups were sensitized by smearing 100 μL of 5% DNCB solution (the solute was a 3:1 mixture of acetone and olive oil), and the sensitization was strengthened again the next day. The blank group was replaced by acetone/olive oil. Then, on the 8th, 12th, and 16th days, 20 μL of 0.5% DNCB was applied to the left ear of mice for secondary sensitization, and the same volume of acetone/olive oil solution was applied to the right ear as a control. The DEX, BBH, BBH-L, BBH-Gel, and BBH-L-Gel groups were given drugs on the fourth day, while the blank group and model group were treated with PBS, with the dosage of 0.1 g/cm^2^ for 12 days. The weight of the mice was weighed and recorded every day, and the skin thickness of the mice was measured with a vernier caliper. Eczema, a common inflammatory skin condition, is often induced by the haptan 2,4-dinitrochlorobenzene (DNCB). The application of a high-concentration solution of DNCB to the skin of mice will result in its combination with the keratin of the mouse skin to form a complete antigen, which in turn stimulates the proliferation of lymphocytes into sensitized lymphocytes. Subsequently, the application of a lower concentration of the DNCB solution to the ear of mice for secondary sensitization will lead to a delayed hypersensitivity reaction after 24 h, exhibiting inflammatory symptoms. With repeated sensitization, the inflammatory response will peak, resulting in ear thickening and the infiltration of inflammatory cells as inflammation markers.

#### 3.6.2. Eczema Area Severity Index (EASI) Evaluation

According to the severity index of clinical eczema [54], the injury degree of the back skin in each group was evaluated. Scores were made according to the damage degree of erythema, edema, papule, exudation, and scab. The severity of each symptom was scored on a scale of 0 (none), 1 (mild), 2 (moderate), and 3 (severe), and a score of 0.5 can be recorded between various symptom scores. The total score of skin lesions in each mouse was added up.

#### 3.6.3. Scratching Behavior of Mice

The scratching behavior of the mice was observed after the last administration of the drug, and the number of times the mice scratched over a period of 20 min was recorded. The scratching time of each group of mice was averaged.

#### 3.6.4. Measurement of Ear Swelling

After the final dose administration, along with fasting but not water deprivation for 12 h, the mice were euthanized with an intraperitoneal injection of a 20% urethane solution. The ear samples, liver, spleen, kidney, and back skin of the mice were collected for subsequent analysis. The left and right ears of the mice were excised, and the samples were taken from the same position of the ears with a 6 mm diameter punch. Subsequently, they were quickly weighed on a precision analytical balance, and the ear swelling was calculated (ear swelling = weight of left ear − weight of right ear).

#### 3.6.5. Weight Ratio of the Spleen to the Body

After the mice were euthanized, the spleens were completely isolated and weighed, and the spleen weight (mg)/body weight ratio (g) was calculated.

#### 3.6.6. Effect of BBH-L-Gel on MDA

Skin and liver tissues were taken and weighed, and nine-times saline was added and homogenized to obtain the organ homogenate suspension. The skin and liver homogenates containing 10% supernatant were removed by centrifugation at 6000 r/min for 15 min at a low temperature. In total, 1 mL of tissue homogenate was taken, and 1 mL of TBA (0.67%) solution and 2 mL of TCA (10%) solution were added. It was soaked at 95 °C for 40 min and then taken out, cooled down to room temperature with running water, and centrifuged at 4000 r/min for 8 min, and the absorbance of the supernatant was measured at 532 nm. The degree of tissue oxidation was determined according to the absorbance.

### 3.7. Statistical Analysis

The data were analyzed using SPSS statistics version 23.0, and all data were expressed as the mean ± standard deviation, using one-way ANOVA between multiple groups, where *p* < 0.05 was considered to be a difference and *p* < 0.001 was considered to be a statistically significant difference.

## 4. Conclusions

BBH has a good anti-inflammatory ability and can effectively alleviate inflammatory reactions such as itching and oxidative stress caused by eczema. Due to the low oral bioavailability of BBH, an attempt was made to prepare the liposomes-in-gel for their application in the treatment of skin diseases. Liposome membranes have a similar structure as human cell membranes, allowing the drug to penetrate deep into the skin, and the gel has good adhesion in prolonging the time for liposomes to work on the skin. After the dual investigation of in vitro release and in vivo pharmacodynamics, it was shown that this dosage form could increase the in vitro release of berberine and extend the drug action time. The mouse eczema model was successfully replicated by sensitization with DNCB. Following drug treatment, the redness, scabbing, and exudation of the mice’s back skin significantly reduced, as observed in the skin histopathological sections showing alleviated capillary hyperplasia and the infiltration of inflammatory cells. The ear swelling and spleen index induced by eczema exhibited a declining trend. The BBH-L-Gel demonstrated a good anti-inflammatory effect, alleviating eczema-induced itching and showing similar efficacy to that of the positive control DEX group. However, glucocorticoid drugs have certain side effects, while BBH-L-Gel provides a certain basis for the clinical treatment of eczema using traditional Chinese medicine ingredients. In conclusion, the liposomes-in-gel, as a new dosage form for skin administration, have good biocompatibility with the skin, improve the permeability of the drug, enhance the skin retention of the drug, make the drug release slow, and play a long-term role, which provides a new strategy for the application of berberine hydrochloride to treat eczema in the skin.

## Data Availability

Data are contained within the article.

## Abbreviations

BBH: berberine hydrochloride; BBH-L: berberine hydrochloride-liposomes; BBH-L-Gel: berberine hydrochloride liposomes-gel; BBH-Gel: berberine hydrochloride-gel; B-L: blank liposomes; B-L-Gel: blank liposomes-gel; B-Gel: blank gel; DPPH: 2,2-diphenyl-1-picrylhydrazyl; MDA: malondialdehyde; H_2_O_2_: peroxide; NO: nitric oxide; DNCB: 2,4- dinitrochlorobenzene; IFN-γ: interferon-γ; MAPK: mitogen-activated protein kinase; TNF-α: tumor necrosis factor-α; NF-κB: nuclear factor kappa-B; IL-1β: interleukin-1 beta; IL-6: interleukin-6; IL-17: interleukin-17; IL-31: interleukin-31; PBS: phosphatic buffer solution; AR: analytical reagent; TBA: thiobarbituric acid; TCA: trichloroacetic acid; DEX: dexamethasone; EASI: eczema area severity index; ANOVA: analysis of variance; SP: substance P; SDS: sodium dodecyl sulfate; ROS: reactive oxygen species.
